# URI1 suppresses irradiation-induced reactive oxygen species (ROS) by activating autophagy in hepatocellular carcinoma cells

**DOI:** 10.7150/ijbs.55689

**Published:** 2021-07-22

**Authors:** Yue Xu, Yuan Ji, Xiang Li, JiaZheng Ding, LinQi Chen, YaFeng Huang, Wenxiang Wei

**Affiliations:** 1 Department of Cell Biology, Institute of Bioengineering, School of Medicine, Soochow University, Suzhou 215123, China; 2 Department of Endocrinology, Children's Hospital affiliated to Soochow University, Suzhou, 215000, China

**Keywords:** URI1, autophagy, ROS, HCC, irradiation insensitivity

## Abstract

Radiotherapy has been extensively applied in cancer treatment. However, this treatment is ineffective in Hepatocellular carcinoma (HCC) due to lack of radiosensitivity. Unconventional prefoldin RPB5 interactor 1 (URI1) exhibits characteristics similar to those oncoproteins, which promotes survival of cancer cells. As a consequence of the irradiation, the levels of endogenous reactive oxygen species (ROS) rise. In the current study, we analyzed the role of URI1 in the control of ROS levels in HepG2 cells. Upon URI1 overexpression, HepG2 cells significantly suppressed irradiation-induced ROS, which may help cells escape from oxidative toxicity. And our data demonstrated that overexpression of URI1 not only resulted in an increase of autophagic flux, but also resulted in an further increased capacity of autophagy to eliminate ROS. It indicated that URI1 suppressed irradiation-induced ROS through activating autophagy. Moreover, URI1 activated autophagy by promoting the activities of AMP-activated protein kinase (AMPK). Results showed that overexpression of URI1 increased the phosphorylation of AMPKα at the Thr172 residue and the activated-AMPK promoted the phosphorylation of forkhead box O3 (FOXO3) at the Ser253 residue, which significantly induced autophagy. Taken together, our findings provide a mechanism that URI1 suppresses irradiation-induced ROS by activating autophagy through AMPK/FOXO3 signaling pathway. These new molecular insights will provide an important contribution to our better understanding about irradiation insensitivity of HCC.

## Introduction

Radiotherapy, also known as ionizing irradiation (IR) 1, remains an important component of cancer treatment 2. There are approximately 50% of all cancer patients receiving radiotherapy during their course of illness 3. Radiotherapy is substantially harmful to cancer cells and simultaneously induces DNA damage, cell cycle arrest and apoptosis 4. Hepatocellular carcinoma (HCC) has been one of the most common malignancy and can cause major cancer deaths 5. At present, surgery combined with radiotherapy and chemotherapy is still the mainstream treatment of HCC 6. However, due to the high metastatic rate of cancer cells and tolerance to chemotherapeutic drugs and irradiation, the prognosis of patients after treatment is rather poor 7. Therefore, knowledge of the mechanism that how HCC cells maintain homeostasis and escape from irradiation-induced injury contributes to finding new radiosensitizers and improving the radiosensitivity of liver cancer.

Unconventional prefoldin RPB5 interactor 1 (URI1), also known as RNA polymerase II subunit 5 (RPB5)-mediating protein (RMP) 8, which has been proved highly expressed in multiple tumors and considered as an oncogene 9. It has been reported that URI1 could promote liver tumorigenesis by inhibiting the synthesis of de novo NAD+ 10. Growing evidences supported that the expression of URI1 is increased during irradiation 11 and overexpression of URI1 results in an enhanced survival of HCC cells by inhibiting apoptosis 12. Therefore, in this study, we focused on exploring whether the increased URI1 is one of the reasons for the irradiation insensitivity of HCC cells and how URI1 works in HCC cells.

It has been well established that generation of reactive oxygen species (ROS) is a key event involved in irradiation-induced biological effects 13. Irradiation exposure triggers the production of ROS and persistent oxidative stress 14. Moreover, irradiation can lead to a dysfunctional mitochondrial status and impair the antioxidative defense system, thus promoting massive accumulation of ROS 15. Excessive ROS leads to significant DNA damage and various cellular responses, including cell cycle arrest, senescence and apoptosis 16. It has been reported that knockout of URI1 in gastric cancer cells results in enhanced oxidative stress and DNA damage when cells was exposed to potassium dichromate 17, which means URI plays a role in maintaining redox homeostasis. Therefore, in this study, we explored whether URI1 has an effect on irradiation-induced ROS to escape from oxidative toxicity and our data demonstrated that URI1 could suppress irradiation-induced ROS.

Autophagy is a highly conserved self-digestion process, which can exert protective functions to eliminate dangerous signals, including ROS, inflammation and metabolic precursors 18. Autophagy plays an important role in tumorigenesis and the development of tumors. On the one hand, autophagy could maintain cellular homeostasis to inhibit the canceration of normal cells 19. On the other hand, autophagy could promote the development of tumor by providing nutrient substance which was produced by autophagy degradation 20. Recent studies showed that the resistance of malignancies to irradiation is associated with the activation of cellular autophagy 21. Therefore, in this study, we explored the effect of URI1 on autophagy and whether URI1 suppress irradiation-induced ROS by regulating autophagy to maintain redox homeostasis. Our data demonstrated that autophagy plays an important role in decreasing ROS and URI1 suppresses irradiation-induced ROS by promoting autophagy.

There are multiple signaling molecules or pathways that have been shown to regulate autophagy 22. Forkhead box O3 (FOXO3), a transcription factor, is an important component of complex signaling cascades which control autophagy 23. Increasing evidence showed that the transcription of autophagy-related genes could be promoted when FOXO3 is activated 24. As an upstream of FOXO3, AMP activated protein kinases (AMPK) is the major energy-sensing kinase in the cell and responds to intracellular AMP/ATP levels to regulate a variety of cellular processes, including autophagy and cellular redox homeostasis 25. Therefore, we explored whether URI1 activates autophagy by regulating the activities of FOXO3 and AMPK. Results showed that URI1 activates autophagy by promoting the phosphorylation of AMPKα at the Thr172 residue and the phosphorylation of FOXO3 at the Ser253 residue, indicating that URI1 activates autophagy through AMPK/FOXO3 signaling pathway.

## Materials and methods

### Antibodies and reagents

The following antibodies were used: rabbit anti-MAP1LC3B (3868), rabbit anti-phospho AMPK alpha (Thr172) (2535) and rabbit anti-phospho FOXO3 Ser318/321 (9465) were obtained from CST (Cell Signaling Technology). Mouse anti-URI1 from Santa Cruz Biotechnology (sc-376011). Rabbit anti-FOXO3A (ab109626), rabbit anti-AMPK alpha (ab207442) and rabbit anti-phospho FOXO3 Ser253 (ab154786) were obtained from Abcam.

Methyladenine (3-MA), a class Ⅲ phosphoinositol 3-kinase (PI3K) inhibitor and used as a selective inhibitor of autophagy, was obtained from Selleck. Rapamycin (rapa), a potent and specific mTOR inhibitor and used as an autophagy activator, was obtained from Selleck. Acadesine (AICAR), an adenosine analog and used as an AMPK activator, was obtained from MCE. Doxorubicin hydrochloride (DOX), a cytotoxic anthracycline antibiotic, is an anti-cancer chemotherapy agent. DOX reduced basal phosphorylation of AMPK and its downstream target acetyl-CoA carboxylase, was used as a inhibitor of AMPK. Hydroxy-chloroquine (HCQ), a lysosomal inhibitor to block the fusion of autophagosomes and lysosomes, was used to analyze the cellular autophagic flux, was obtained from Sigma-Aldrich (St Louis, MO, USA). N-Acetyl-L-cysteine (NAC), was used as an antioxidant and obtained from Sigma-Aldrich (St Louis, MO, USA). All other chemicals were obtained from Sigma-Aldrich (St Louis, MO, USA) unless mentioned elsewhere.

### Plasmids, cell culture and transfection

The following cells were used in this study: HepG2 (a human hepatoma cell line), stable cell lines pCDNA3.1-URI1-HepG2, pGPU6-URI1i-HepG2 were saved in our lab. The plasmid pGPU6-Neo was purchased from Jima Co (Shanghai, China). The plasmid pGPU6-URI1i for URI1 depletion and pCDNA3.1-URI1 for URI1 overexpression. The plasmid pCDNA3.1-EGFP-LC3 was saved in our lab.

Cells were routinely cultured in Dulbecco's Modified Eagle Medium (DMEM), supplemented with 10% fetal bovine serum (FBS) (Sino-American Biotechnology Co, Shanghai, China) and 1% penicillin-streptomycin (Beyotime Biotech, Shanghai, China) at 37℃ in an incubator with 5% CO_2_.

Cells were initially seeded in a 6-well plate at a density of 4×10^5^ cells per well in 2 ml culture media. After overnight incubation, cells were transfected with Lipofectamine 3000 (ThermoFisher, American) according to the manufacturer's instructions. Cells were transfected with plasmids pGPU6-URI1i for URI1 depletion (URI1i) and pCDNA3.1-URI1 for URI1 overexpression (URI1o). Then transfected cells were cultured at 37℃ in a humidified incubator containing 5% CO_2_.

### Irradiation procedure

Cells were cultured in 6-well plate and moved to the irradiation room when the cells reached 90% confluence. The cells were subjected to Irradiation (IR) at a dosage of 10 Gy and a dose rate of 2Gy/min. The cells were then placed back in the incubator for continuous culturing before the samples were collected for further analysis.

### Analysis of intracellular ROS

The ROS assay kit (Beyotime Biotech, Shanghai, China) was used to assess intracellular ROS levels. Cells were incubated with 10 mM of DCFH-DA at 37℃ for 30 minutes, gently washed with PBS for 3 times, and then imaged under a fluorescence microscope or analyzed by flow cytometry and microplate reader. To determine the fluorescence intensity, the incubated cells were measured on a flow cytometer with the following parameters: λ ex = 640 nm and λ em = 675 nm. The results are shown as images and fluorescence intensity.

### Western blot analysis

The cell were washed with PBS and subsequently lysed in lysis buffer (RIPA with protease and phosphatase inhibitor cocktails) on ice. The concentrations of proteins were detected using a BCA protein assay kit (Beyotime Biotech, Shanghai, China). The proteins were separated on SDS-polyacrylamide gels and transferred to PVDF membranes. Membranes were blocked with 5% nonfat milk in Tris-buffered saline for 1.5 h at room temperature and washed with Tween 2.0 in Tris-buffered saline (TBST), then incubated overnight at 4℃ with the indicated primary antibody against MAP1LC3B (1:1000), which was used as a marker for autophagy. Other antibodies used in this study include AMPKα (1:1000), p-AMPKα(Thr172) (1:1000), URI1 (1:800), FOXO3A (1:2000), p-FOXO3A(Ser253) (1:1000) and p-FOXO3A(Ser318/321) (1:2000). Rabbit GAPDH antibody (1:2000) was used as control. After washing thrice with TBST, the membranes were blotted with the respective secondary antibody for 2 hour at room temperature. The band density was analyzed using Image J 1.80 software.

### Confocal microscopic analysis

Cells were cotransfected with enhanced green fluorescent protein (EGFP)-LC3 expression plasmid and pCDNA3.1-URI1 plasmid by using Lipofectamine 3000 (ThermoFisher, American) according to the manufacturer's instructions. After 36 h, cells were treated with HCQ (50μM) for 12h. The cells were washed with PBS on ice and fixed with 4% paraformaldehyde solution. Following permeabilization with 0.5% Triton X-100 for 15 min, the cells were incubated with goat serum for 1 h and counterstained with DAPI for 5 min. The confocal images were captured using an A1 Confocal Laser Microscope System (Nikon Corp, Tokyo Japan).

### RT-PCR

Total RNAs were extracted using Trizol (Invitrogen, American) and reverse transcription was performed from 2.5 μg total RNAs using the All-in-One cDNA Synthesis SuperMix (Bimake, American). The abundance of mRNA was detected by an ABI prism 7500 system with 2×SYBR Green qPCR Master Mix (bimake, American). The quantity of mRNA was calculated using the ΔΔCt method and GAPDH were used as a control. All reactions were performed as triplicates.

The following primers were used in this study: GAPDH; forward (fwd) 5′-CGACCACTTTGTCAAGCTCA-3′, reverse (rev) 5′-AGGGGAGATTCAGTGTGGTG-3′; MAP1LC3B; fwd 5′-CGATACAAGGGTGAGAAGCA-3′, rev 5′-CCTCTGAGATTGGTGTGGAG-3′; P62; fwd 5′-CCAGAGAGTTCCAGCACAGA-3′, rev 5′-CCCTACAGATGCCAGAATCC-3′; ATG5; fwd 5′-GGCTGAGTGAACATCTGAGC-3′, rev 5′-GCCCAGTTGCCTTATCTGA-3′; ATG12 fwd 5′-TCAGTCCTTTGCTCCTTCC-3′, rev 5′-TTTCAACCTTGGAGGCAGAT-3′; FOXO3; fwd 5′-AGTCTCCTGTCAGCCAGTCTAT-3′, rev 5′-TCTGTTCCAAGGGTAAGTGC-3′.

### Statistical analysis

All data in the current study were presented as the Mean ± SD of 3 independent experiments. Data comparisons among different groups were performed using Student's t tests or one-way analysis of variance (ANOVA) in SPSS (version 20, Chicago, IL, USA), and p ≤ 0.05 was deemed statistically significant.

## Results

### URI1 suppresses irradiation-induced ROS in HCC cells

**Figure 1:**
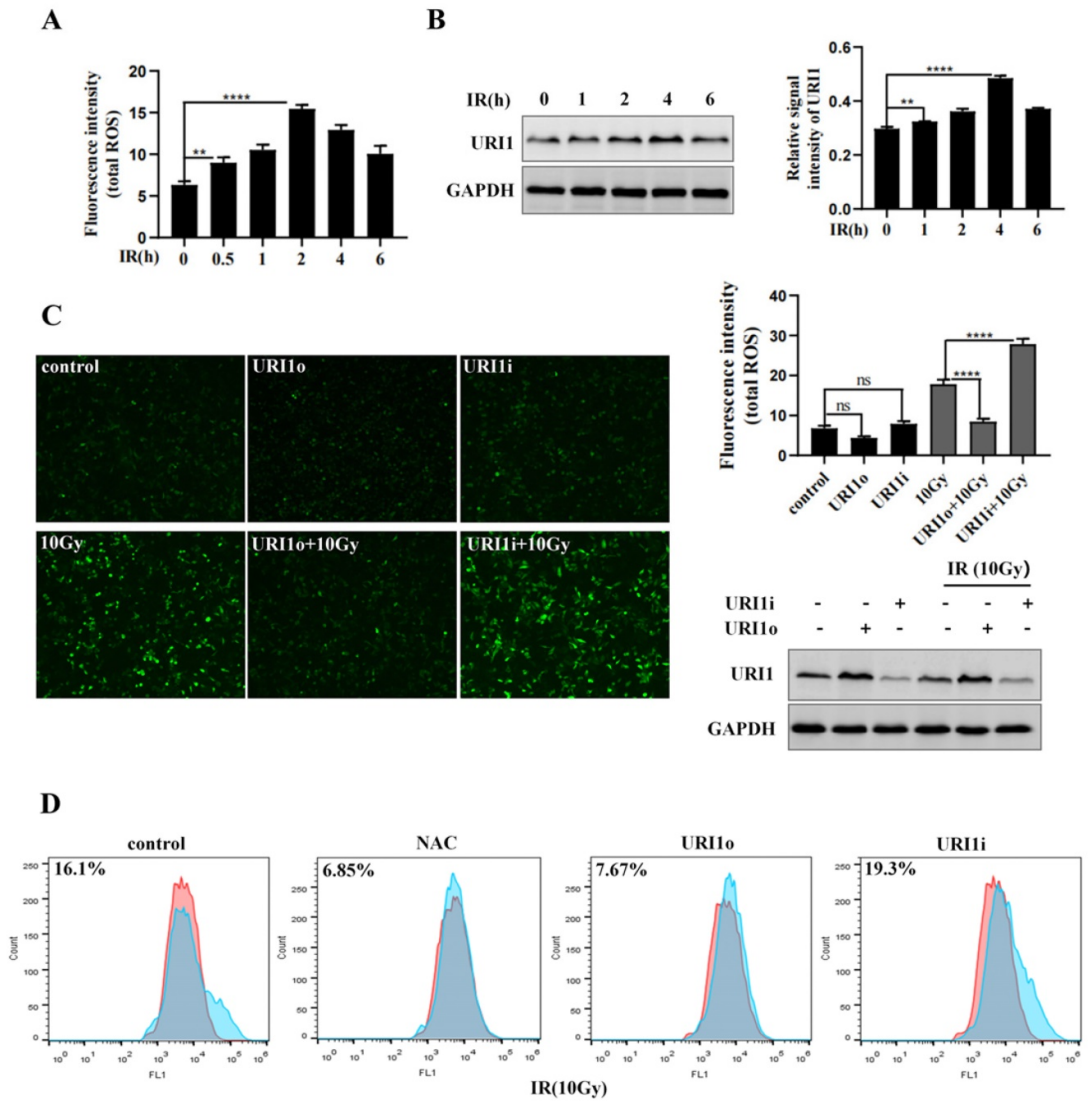
URI1 suppresses irradiation-induced ROS in HCC cells. **(A)** Irradiation (IR) induces ROS production. HepG2 cells were exposed to 10Gy of ^60^Coγ irradiation for different times and ROS levels were analyzed by microplate reader. Values are representative results of 3 independent experiments. **p<0.005, ****p<0.0001. **(B)** Irradiation induces URI1 expression in HepG2 cells. HepG2 cells were exposed to 10Gy of ^60^Coγ irradiation for different times. Left panel: URI1 was analyzed by western blot. GAPDH was used as a loading control. Right panel: The ratio of URI1 to GAPDH was quantified by using Image J 1.80 software. Representative results of 3 independent experiments are shown. **p<0.005, ****p<0.0001. **(C)** URI1 suppresses irradiation-induced ROS in HepG2 cells. Left panel: HepG2 cells were transfected with URI1 overexpression (URI1o) or URI1 interference (URI1i) plasmid followed by 2 h treatment with irradiation and ROS levels were analyzed by fluorescence microscopy (original magnification 400x). Images are representative of 3 independent experiments. Right upper panel: Intensity of ROS fluorescence was quantified by using Image J 1.80 software. Values are representative results of 3 independent experiments. ****p<0.0001. Right lower panel: Western blot showed URI1 expression level after HepG2 cells were transfected with URI1o or URI1i plasmid. **(D)** Depletion of URI1 increases irradiation-induced ROS in HepG2 cells. ROS levels were measured by flow cytometry and normalized by comparing the fluorescence representing ROS levels in different cells. NAC was used as an inhibitor of ROS.

Many studies reported that irradiation (IR) induces reactive oxygen species (ROS) production, resulting in oxidative damage 26. We examined the induction of ROS by irradiation firstly. Results showed that ROS levels reached the highest degree at 2 h, and then showed a decreasing trend (Figure 1A). As the expression of URI1 was increased by irradiation 11, we examined the expression of URI1 for different times during irradiation. Results showed that the expression of URI1 reached maximum at 4 h, while declined at 6 h (Figure 1B).

To explore whether the upregulation of URI1 was a cause of ROS reduction after exposing irradiation for 2 h, HepG2 cells were transfected with URI1 overexpression (URI1o) plasmid or URI1 interference (URI1i) plasmid and then subjected to irradiation to induce ROS. Results showed that the ROS in URI1 overexpressing cells (URI1o) was significantly lower than that of control (Figure 1C). On the contrary, the ROS in URI1 interfering cells (URI1i) was higher than that of control (Figure 1C). To further confirm these results, ROS was analyzed by flow cytometry. As expected, overexpression of URI1 suppressed irradiation-induced ROS and depletion of URI1 elevated irradiation-induced ROS (Figure 1D). Taken together, these data indicated that URI1 played an important role in suppressing irradiation-induced ROS in HepG2 cells.

### URI1 induces autophagy in HCC cells

**Figure 2:**
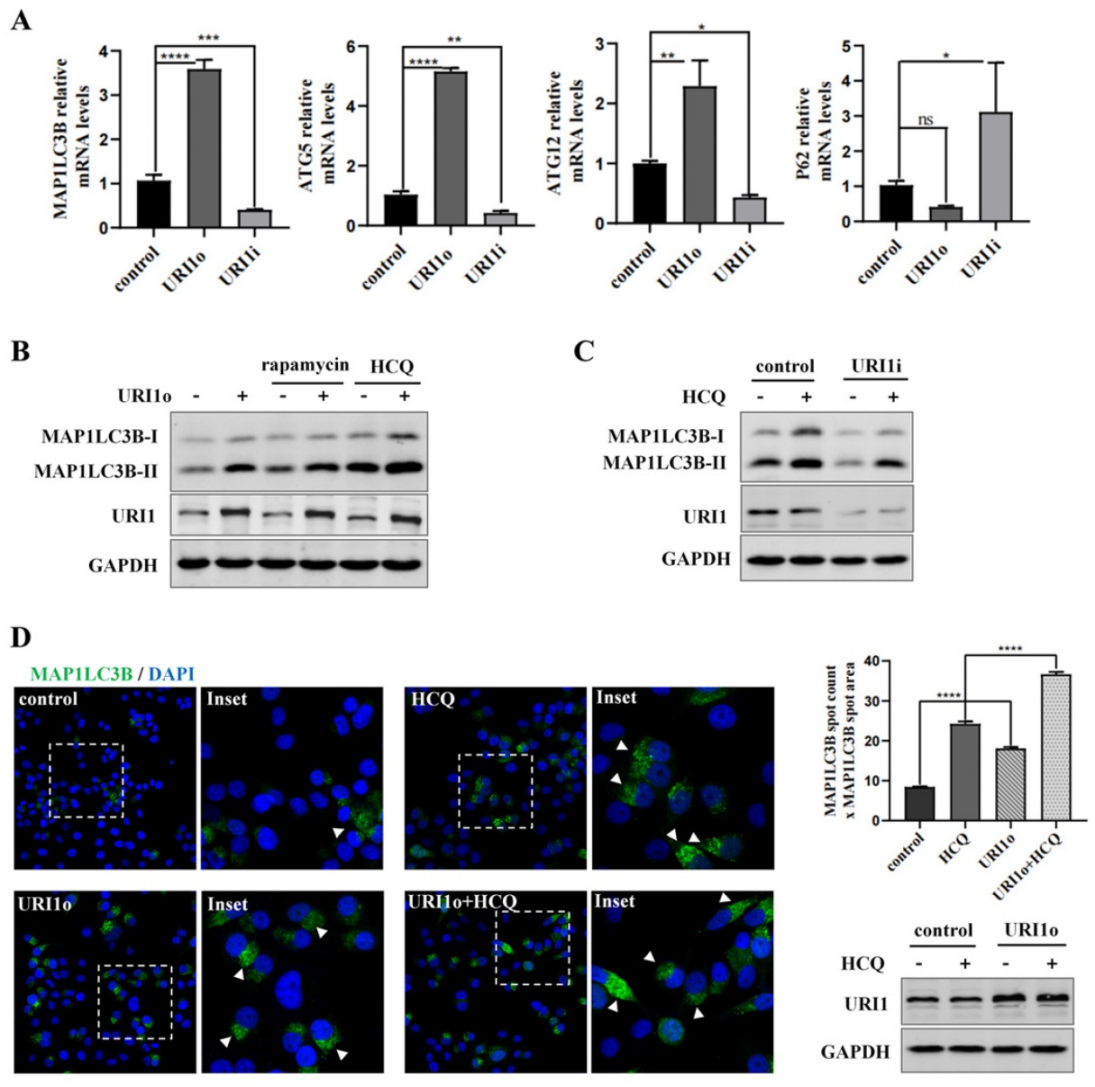
URI1 induces autophagy in HCC cells. **(A)** URI1 induces the mRNA expression of autophagy-related genes in HepG2 cells. HepG2 cells were transiently transfected with URI1 overexpression (URI1o) or URI1 iterference (URI1i) plasmid and the mRNA expression of autophagy-related genes were analyzed by qRT-PCR. Data of 3 independent experiments are presented as mean ± SEM. *p<0.05, **p<0.005, ***p<0.001, ****p<0.0001. **(B)** URI1 induces autophagy in HepG2 cells. HepG2 cells were transiently transfected with URI1o plasmid. After 36 h, the cells were treated with rapamycin (10μM) for 9 h or HCQ (50μM) for 12 h. MAP1LC3B-II was analyzed by western blot. GAPDH was used as a loading control. HCQ was used as an inhibitor of autophagy and rapamycin was used as an agonist of autophagy. **(C)** Depletion of URI1 reduces autophagic flux in HepG2 cells. HepG2 cells were transfected with URI1i plasmid. After 36 h, the cells were treated with HCQ (50μM) for 12 h. MAP1LC3B-II was analyzed by western blot. GAPDH was used as a loading control. **(D)** URI1 induces formation of MAP1LC3B positive autophagosomes. Control: HepG2 cells were transfected with EGFP-LC3 plasmid alone. HCQ: HepG2 cells were transfected with EGFP-LC3 plasmid followed by HCQ (50μM) treatment for 12 h. URI1o: HepG2 cells were cotransfected with EGFP-LC3 and URI1o plasmids. URI1o+HCQ: HepG2 cells were cotransfected with EGFP-LC3 and URI1o plasmids followed by HCQ (50μM) treatment for 12 h. Left panel: Representative pictures showed MAP1LC3B in green and DAPI positive nuclei in blue. Arrow heads indicated the MAP1LC3B positive autophagosomes. Right upper panel: Array scan quantification based on spot count and area of the spot. Quantification of data from 3 independent experiments performed is shown as mean ± SEM. ****P<0.0001. Right lower panel: Western blot showed URI1 expression level after HepG2 cells were transfected.

Many studies showed that autophagy has a crucial role in eliminating ROS to maintain redox homeostasis 27. To investigate the effect of URI1 on cellular autophagy in HepG2 cells, the mRNA expression of autophagy-related genes was determined. Expression of microtubule-associated protein 1 light chain 3β (MAP1LC3B), autophagy-related gene 5 (ATG5) and autophagy-related gene 12 (ATG12) was all significantly upregulated when cells were transfected with URI1 overexpression (URI1o) plasmids. Meanwhile, Sequestosome 1 (P62), a substrate of autophagy, which was used as a reporter of autophagy activity 28, was relatively downregulated when URI1 was overexpressed (Figure 2A). The results showed an opposite trend when URI1 was depleted (Figure 2A). To further determine the effect of URI1 on autophagy, an agonist of autophagy (rapamycin) was added after the transfection. MAP1LC3-II, a lipidated form of MAP1LC3B-I ubiquitin-like protein, which associates with autophagosomal membranes and is used as a marker of autophagy 29. Western blot of the MAP1LC3-II showed that rapamycin stimulated the cellular autophagy and overexpression of URI1 increased rapamycin-induced autophagy (Figure 2B). An increase of MAP1LC3-II does not represent an increase in autophagic flux, since it can also indicate an inhibition of autophagosomes clearance 30. HCQ, a lysosomal inhibitor, can block the fusion of autophagosomes and lysosomes, resulting in degradation inhibition of autophagosomes and accumulation of MAP1LC3B-II 31. Therefore, HCQ was used to analyze the effect of URI1 on autophagic flux. Results showed that overexpression of URI1 increased HCQ-induced accumulation of MAP1LC3B-II (Figure 2B), and depletion of URI1 decreased HCQ-induced accumulation of MAP1LC3B-II (Figure 2C). These results indicated that URI1 promoted the autophagic flux in HepG2 cells. To investigate whether URI1 also promoted autophagosomes formation, the number (spot count) and size (spot area) of MAP1LC3B-positive vesicles were analyzed by fluorescence microscopy in HepG2 cells cotransfected with URI1 overexpression (URI1o) plasmid and EGFP-LC3 plasmid followed by HCQ treatment. Results showed that URI1 increased the number and size of MAP1LC3B-positive punctate, indicating the increased formation of autophagosomes (Figure 2D).

### URI1 suppresses irradiation-induced ROS by promoting autophagy

**Figure 3:**
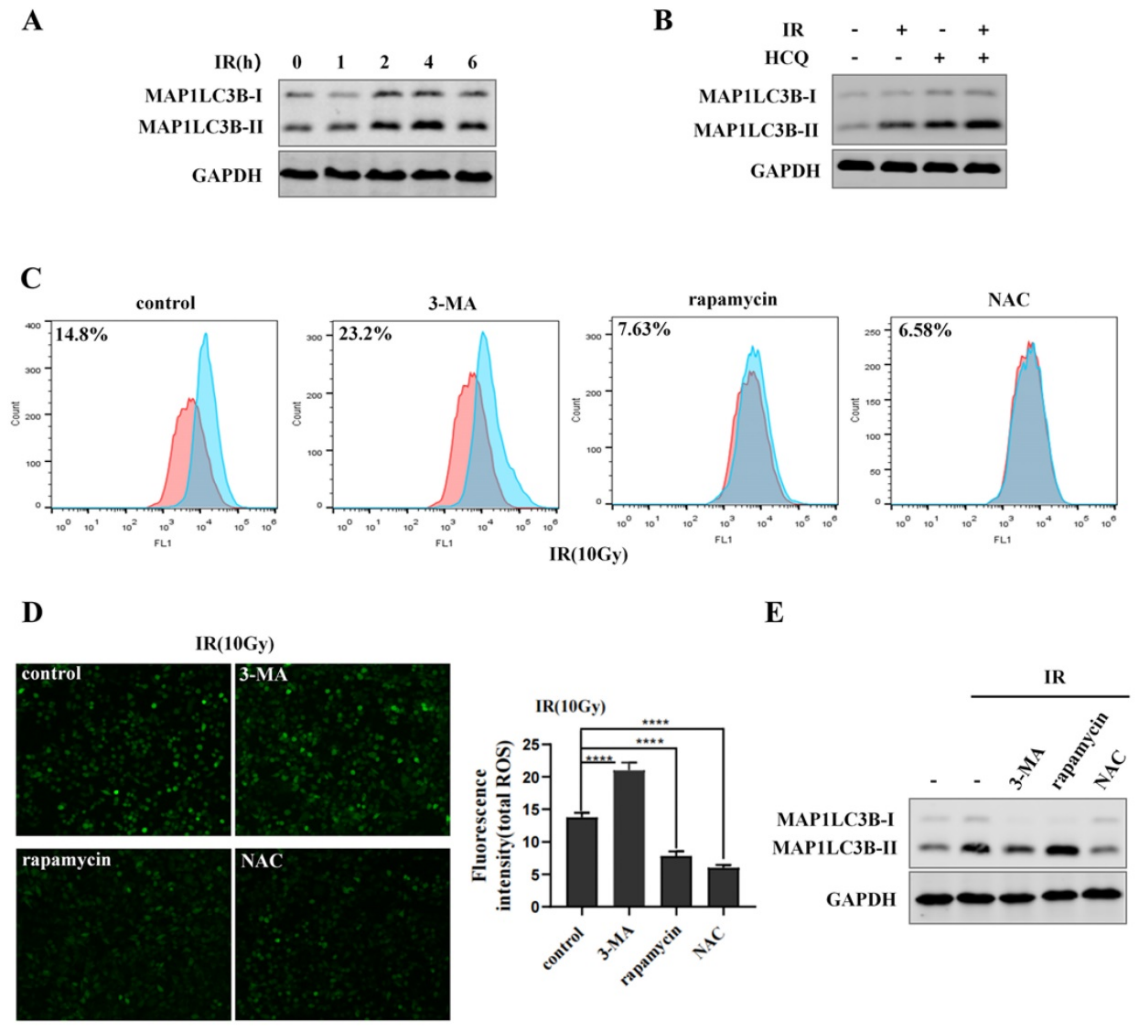
Autophagy eliminates irradiation-induced ROS. **(A)** Irradiation (IR) induces the MAP1LC3-II expression in HepG2 cells. HepG2 cells were exposed to irradiation (10Gy of ^60^Coγ) for different times. MAP1LC3-II was analyzed by western blot. GAPDH was used as a loading control. **(B)** Irradiation promotes the autophagic flux in HepG2 cells. HepG2 cells were pre-treated with HCQ (50μM) for 12 h under the condition of irradiation. MAP1LC3B-II was analyzed by western blot. GAPDH was used as a loading control. HCQ was used as an inhibitor of autophagy. **(C)** Autophagy eliminates irradiation-induced ROS. HepG2 cells were treated with 3-MA (2.5mM) for 6 h or treated with rapamycin (10μM) for 9 h under the condition of irradiation. 3-MA was used as an inhibitor of autophagy and rapamycin was used as an agonist of autophagy. NAC was used as an inhibitor of ROS. ROS levels were analyzed by flow cytometry and normalized by comparing the fluorescence representing ROS levels in different cells. **(D)** ROS levels in **(C)** were measured by fluorescence microscopy (original magnification 400x). Images are representative of 3 independent experiments. Right panel: Intensity of ROS fluorescence was quantified by using Image J 1.80 software. Values are representative results of 3 independent experiments. ****p<0.0001. **(E)** The autophagy of cells which treated with 3-MA or rapamycin in combination with irradiation were reflected by western blot. GAPDH was used as a loading control.

Mitochondria are the major source of reactive oxygen species (ROS) in most cells 32. Excessive ROS can function as a destructive molecule, which contributes to genomic instability, cell death or tumorigenesis 33. Many researches have previously demonstrated that autophagy can eliminate damaged mitochondria and toxic aggregates 34. To determine the effect of irradiation on autophagy, cells were exposed to 10Gy of ^60^Coγ irradiation (IR) and MAP1LC3-II was analyzed by western blot. Results showed that MAP1LC3-II was increased after irradiation and reached maximum at 4 h, while declined at 6 h (Figure 3A). Then, cells were exposed to irradiation with or without HCQ pre-treatment. Results showed that cells treated with irradiation and HCQ showed a significant increase of MAP1LC3B-II accumulation compared to the cells exposed to irradiation alone (Figure 3B), indicating that irradiation promoted autophagic flux in HepG2 cells. To demonstrate the effect of autophagy on ROS during irradiation, cells were treated with 3-MA (an inhibitor of autophagy) or rapamycin (an agonist of autophagy) and ROS was determined by fluorescence microscope and flow cytometry. HepG2 cells treated with 3-MA and irradiation showed an increase in ROS levels compared to the cells exposed to irradiation alone, indicating that irradiation-induced ROS could not be eliminated when autophagy was inhibited by 3-MA (Figure 3C&D). Likewise, ROS was obviously abated when cells treated with rapamycin and irradiation compared to the cells exposed to irradiation alone, indicating that irradiation-induced ROS could be eliminated when autophagy was activated by rapamycin (Figure 3C&D). The autophagy of cells treated with 3-MA or rapamycin in combination with irradiation was examined by western blot (Figure 3E). These data indicated that autophagy decreased ROS levels in HepG2 cells during irradiation to inhibit oxidative damage.

**Figure 4:**
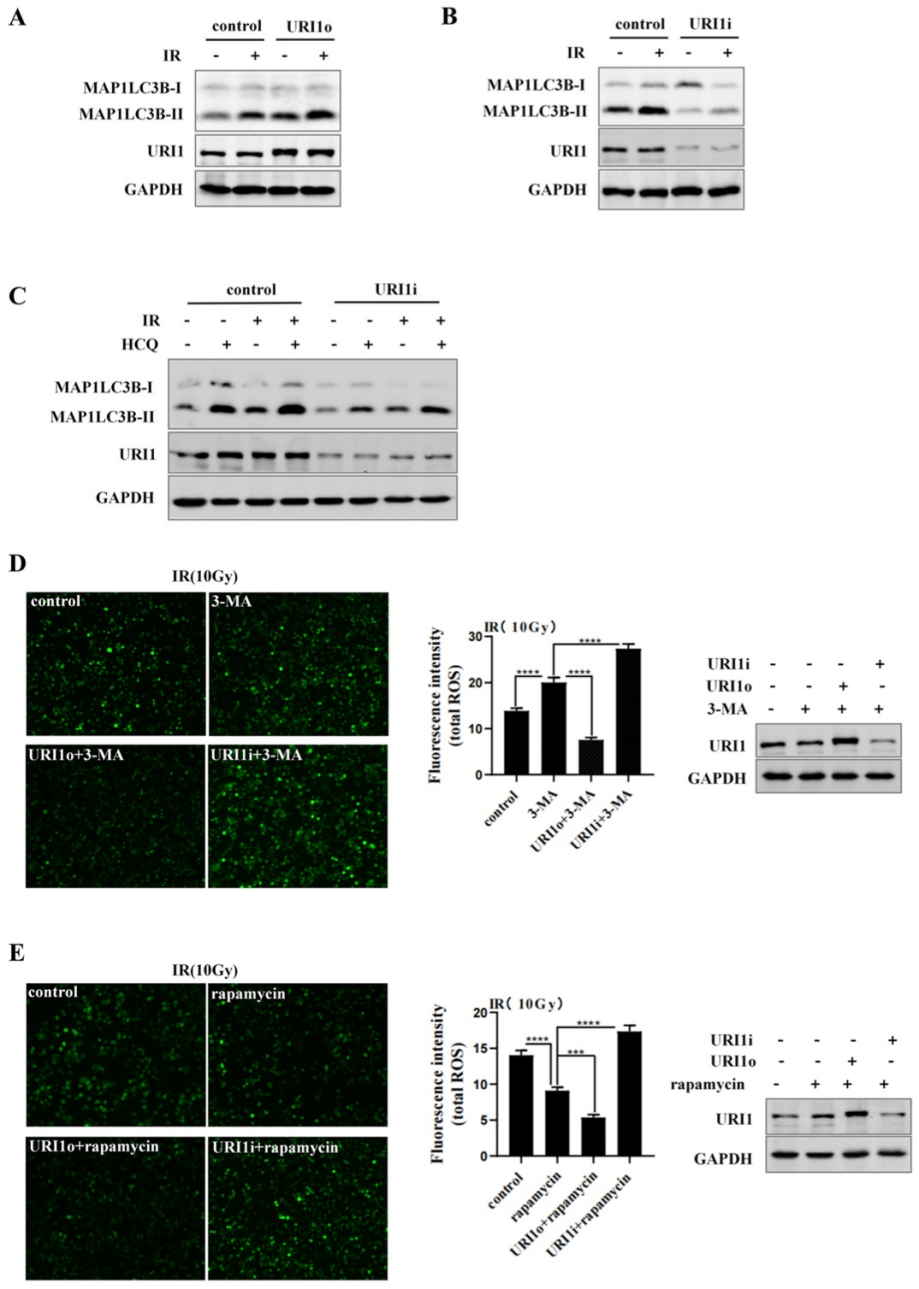
URI1 suppresses irradiation-induced ROS by promoting autophagy. **(A)** overexpression of URI1 increases irradiation-induced MAP1LC3B-II expression in HepG2 cells. HepG2 cells were transfected with URI1 overexpression (URI1o) plasmid and then treated with irradiation (10Gy of ^60^Coγ) for 4 h. MAP1LC3B-II was analyzed by western blot. GAPDH was used as a loading control. **(B)** Depletion of URI1 decreases irradiation-induced MAP1LC3B-II expression in HepG2 cells. HepG2 cells were transfected with URI1 interference (URI1i) plasmid and then treated with irradiation for 4 h. MAP1LC3B-II was analyzed by western blot. GAPDH was used as a loading control. **(C)** Depletion of URI1 inhibits irradiation-increased autophagic flux. HepG2 cells were transfected with URI1i plasmid followed by 12 h treatment with HCQ (50μM) in the presence or absence of irradiation and lysed directly after the treatment. URI1 and MAP1LC3B-II were analyzed by western blot. GAPDH was used as a loading control. **(D)** Depletion of URI1 further increases ROS levels when 3-MA inhibits autophagy. Left panel: HepG2 cells were transfected with URI1o or URI1i plasmid followed by 6 h treatment with 3-MA (2.5mM) and then treated with irradiation for 2 h. The ROS were imaged under a fluorescence microscope. Images are representative of 3 independent experiments. Middle panel: Intensity of ROS fluorescence was quantified by using Image J 1.80 software. Values are representative results of 3 independent experiments. ****p<0.0001. Right panel: Western blot showed URI1 expression level after HepG2 cells were transfected with URI1o or URI1i plasmid. **(E)** Overexpression of URI1 further decreases ROS levels when rapamycin activates autophagy. Left panel: HepG2 cells were transfected with URI1o or URI1i plasmid followed by 9 h treatment with rapamycin (10μM) and then treated with irradiation for 2 h. The ROS were imaged under a fluorescence microscope. Images are representative of 3 independent experiments. Middle panel: Intensity of ROS fluorescence was quantified by using Image J 1.80 software. Values are representative results of 3 independent experiments. ***p<0.001, ****p<0.0001. Right panel: Western blot showed URI1 expression level after HepG2 cells were transfected with URI1o or URI1i plasmid.

We have confirmed that URI1 suppressed irradiation-induced ROS (Figure 1C&D), URI1 induced autophagy (Figure 2B&D) and autophagy decreased irradiation-induced ROS in HepG2 cells (Figure 3B&C). Then, we examined whether URI1 suppressed irradiation-induced URI1 by promoting autophagy in HepG2 cells. Firstly, we examined whether URI1 still induced autophagy under the condition of irradiation in HepG2 cells. Results showed that cells transfected with URI1o plasmid and exposed to irradiation showed a significant increase in MAP1LC3B-II compared to the cells exposed to irradiation alone (Figure 4A). Likewise, cells transfected with URI1i plasmid and exposed to irradiation showed a decrease in MAP1LC3B-II compared to the cells exposed to irradiation alone (Figure 4B). To determine whether depletion of URI1 inhibited autophagic flux under the condition of irradiation, cells were transfected with URI1i plasmid and treated with HCQ in the presence or absence of irradiation. MAP1LC3B-II was analyzed by western blot. Noteworthy, the accumulation of MAP1LC3B-II were significantly decreased in both cases when URI1 was depleted (Figure 4C), indicating that depletion of URI1 not only suppressed the basal autophagy level, but also inhibited irradiation-induced autophagy. To determine whether URI1 suppresses irradiation-induced ROS by promoting autophagy, HepG2 cells were transfected with URI1o or URI1i plasmid followed by treatment with 3-MA or rapamycin, and then exposed to irradiation for 2 h. The results showed that when autophagy was inhibited by 3-MA, depletion of URI1 resulted in a higher level of ROS compared to cells treated with 3-MA alone (Figure 4D). Likewise, when autophagy was activated by rapamycin, overexpression of URI1 resulted in a lower level of ROS compared to the cells treated with rapamycin alone (Figure 4E). Taken together, these data indicated that URI1 suppresses irradiation-induced ROS by promoting autophagy.

### URI1 induces autophagy via FOXO3

**Figure 5:**
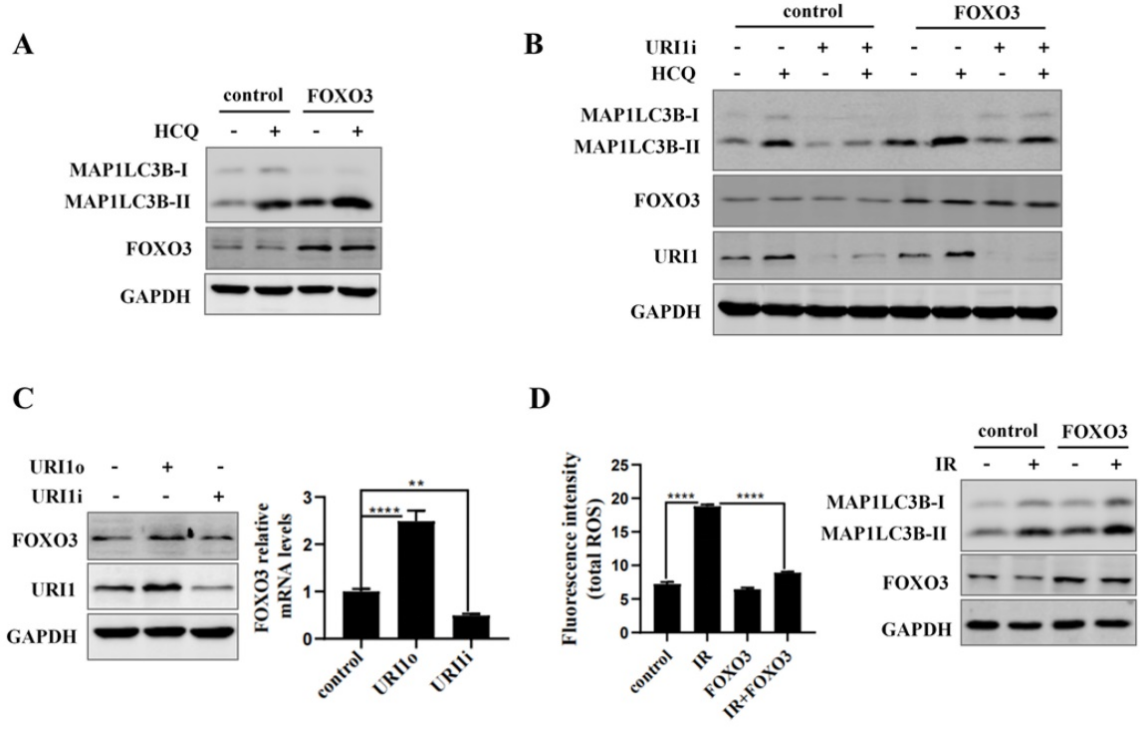
URI1 induces autophagy via FOXO3.** (A)** FOXO3 induces autophagy in HepG2 cells. HepG2 cells were transiently transfected with FOXO3 overexpression plasmid. After 36 h, the cells were treated with HCQ (50μM) for 12 h. MAP1LC3B-II was analyzed by western blot. GAPDH was used as a loading control. **(B)** Depletion of URI1 inhibits FOXO3-induced autophagy. HepG2 cells were transfected with FOXO3 overexpression plasmid followed by 12 h treatment with HCQ (50μM) in the presence or absence of URI1i plasmid and lysed directly after the treatment. URI1, FOXO3 and MAP1LC3B-II were analyzed by western blot. GAPDH was used as a loading control. **(C)** URI1 increases the expression of FOXO3 in both mRNA and protein levels in HepG2 cells. Left panel: HepG2 cells were transfected with URI1 overexpression (URI1o) or URI1 interference (URI1i) plasmid and FOXO3 was analyzed by western blot. GAPDH was used as a loading control. Right panel: HepG2 cells were transfected with URI1o or URI1i plasmid and the mRNA level of FOXO3 was analyzed by qRT-PCR. Data of 3 independent experiments are presented as mean ± SEM. **p<0.005, ****P<0.0001. **(D)** FOXO3 decreases irradiation-induced ROS in HepG2 cells. Left panel: HepG2 cells transfected with FOXO3 overexpression plasmid and then exposed to irradiation for 2 h. ROS levels were analyzed by microplate reader. Values are representative results of 3 independent experiments. ****p<0.0001. Right panel: Western blot showed FOXO3 expression level after HepG2 cells were transfected with FOXO3 overexpression plasmid.

Forkhead box O3 (FOXO3) transcription factor plays an important role in cellular autophagy 35. Many studies reported that FOXO3 defenses against oxidative stress through activating cellular autophagy 36. Therefore we investigated the involvement of FOXO3 in the process of URI1-induced autophagy. Firstly, we examined the effect of FOXO3 on autophagy. Results showed that overexpression of FOXO3 improved HCQ-induced accumulation of MAP1LC3B-II (Figure 5A). To determine whether depletion of URI1 inhibited FOXO3-induced autophagy, cells were cotransfected with FOXO3 overexpression plasmid and URI1i plasmid followed by HCQ treatment. Autophagy was evaluated by MAP1LC3B-II which failed to increase in response to elevated FOXO3 level when URI1 was depleted, indicating that URI1 promoted autophagy via FOXO3 (Figure 5B). To determine whether FOXO3 is implicated in the URI1-induced autophagy, the mRNA and protein levels of FOXO3 were analyzed by qRT-PCR and western blot when URI1 was overexpressed or depleted. Results showed that URI1 significantly increased the expression of FOXO3 in both mRNA and protein (Figure 5C). To investigate the effect of FOXO3 on ROS, HepG2 cells were transfected with FOXO3 overexpression plasmid and then subjected to irradiation. Results showed that cells which FOXO3 was overexpressed showed a significant decrease in ROS levels compared to the cells exposed to irradiation alone (Figure 5D).

### URI1 induces autophagy by activating AMPK

**Figure 6:**
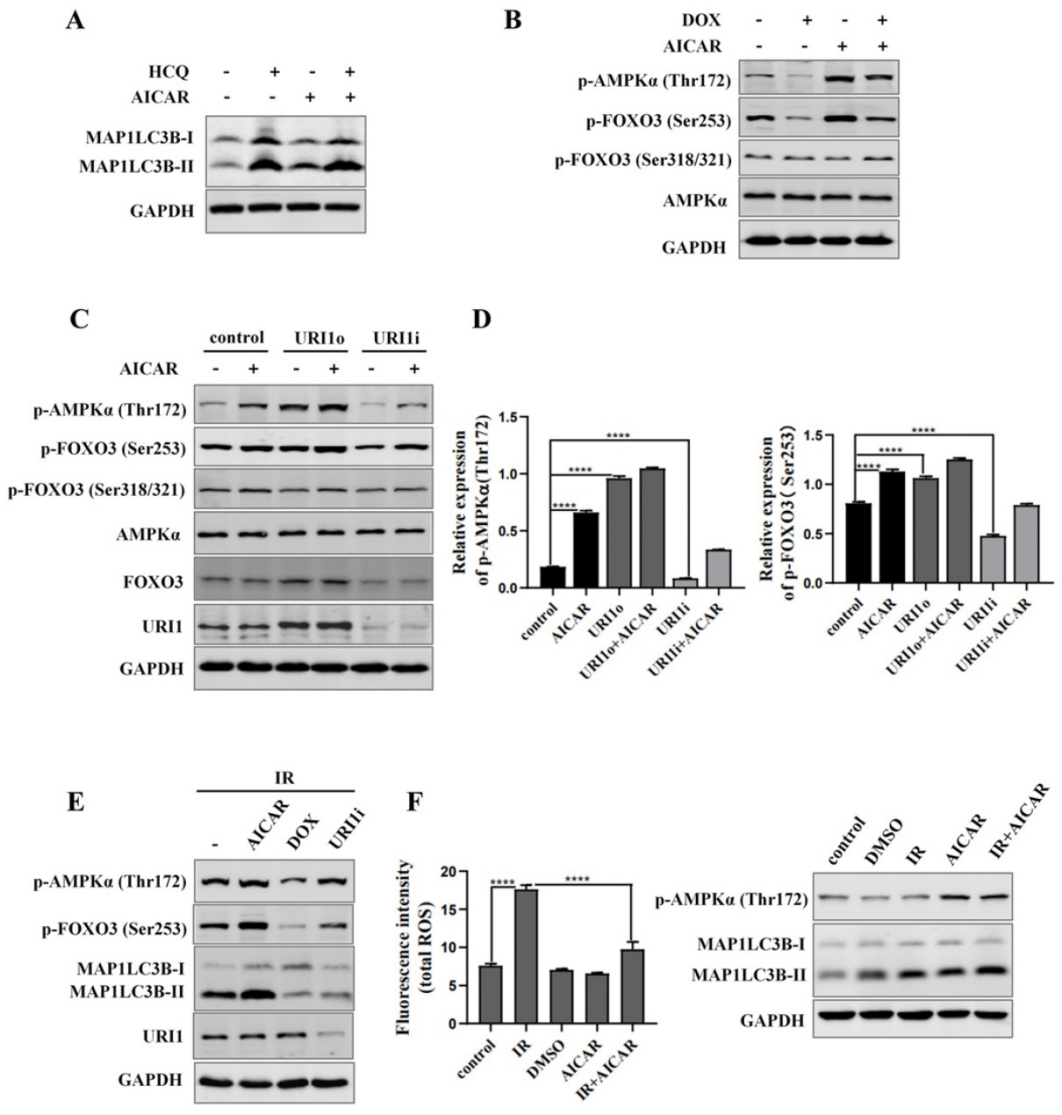
URI1 induces autophagy by activating AMPK. **(A)** Activation of AMPK induces autophagy in HepG2 cells. HepG2 cells were treated with AICAR (1mM) for 6 h and HCQ (50μM) for 12 h. MAP1LC3-II was analyzed by western blot. GAPDH was used as a loading control. AICAR was used as an activator of AMPK. **(B)** Activation of AMPK induces phosphorylation of FOXO3 at the Ser253 residue. HepG2 cells were treated with AICAR (1mM) or DOX (10μM) for 6 h. FOXO3 was analyzed by western blot for p-FOXO3 (Ser253) and p-FOXO3 (Ser318/321). GAPDH was used as a loading control. AICAR was used as an activator of AMPK. DOX was used as an inhibitor of AMPK.** (C)** URI1 induces phosphorylation of AMPK at the Thr172 residue and phosphorylation of FOXO3 at the Ser253 residue. HepG2 cells with stable interference (URI1i) or overexpression (URI1o) of URI1 were treated with AICAR (1mM) for 6 h. AMPK was analyzed by western blot for p-AMPKα (Thr172). FOXO3 was analyzed by western blot for p-FOXO3 (Ser253) and p-FOXO3 (Ser318/321). GAPDH was used as a loading control. **(D)** The ratio of p-AMPKα (Thr172) to AMPKα and the ratio of p-FOXO3(Ser253) to FOXO3 were quantified by using Image J 1.80 software. Representative results of 3 independent experiments are shown. **(E)** Depletion of URI1 decreases the phosphorylation of AMPK and FOXO3 under the condition of irradiation. HepG2 cells were separately treated with URI1i plasmid or AICAR or DOX and then irradiation for 2 h. AMPK was analyzed by western blot for p-AMPKα (Thr172). FOXO3 was analyzed by western blot for p-FOXO3 (Ser253). GAPDH was used as a loading control. **(F)** Activation of AMPK decreases irradiation-induced ROS. HepG2 cells were treated with AICAR (1mM) for 6h followed by irradiation for 2 h. Right panel: ROS were analyzed by microplate reader. Values are representative results of 3 independent experiments. ****p<0.0001. Left panel: Western blot showed the phosphorylation of AMPK.

AMP activated protein kinases (AMPK), an upstream of FOXO3, plays an important role in cellular autophagy 37. Studies proved that AMPK regulates oxidative metabolism via cellular autophagy 38. We examined the effect of AMPK on autophagy in HepG2 cells firstly. Results showed that the HCQ-induced accumulation of MAP1LC3B-II was significantly increased when AMPK was activated by AICAR (an activator of AMPK) (Figure 6A). Phosphorylation of FOXO3 has been shown to promote its transcriptional activity which activates the expression of autophagy-related genes 39. Therefore, the effect of AMPK on phosphorylation of FOXO3 was examined. The results showed that the phosphorylation of FOXO3 at the Ser253 residue was increased when AMPK was activated by AICAR and decreased when AMPK was inhibited by DOX (an inhibitor of AMPK) (Figure 6B). To investigate whether URI1 activates AMPK/FOXO3 pathway under cellular basic condition, the activation of AMPKα were analyzed when URI1 was stably overexpressed (URI1o) or depleted (URI1i). Results showed that the activated form of AMPKα with a phosphorylated Thr^172^ was significantly increased with URI1 stably overexpressed (URI1o) and decreased with URI1 stably depleted (URI1i) (Figure 6C). The phosphorylation of FOXO3 at the Ser253 residue was also significantly increased with URI1 stably overexpressed (URI1o) and decreased with URI1 stably depleted (URI1i) (Figure 6C). It is worth noting that URI1-mediated the phosphorylation of FOXO3 seemed to be specific to the serine 253, as no effect on phosphorylation of Ser318/321 when URI1 was stably overexpressed or depleted (Figure 6C). The ratio of p-AMPKα (Thr172) to AMPKα and the ratio of p-FOXO3 (Ser253) to FOXO3 were markedly higher in URI1o cells compared to the control (Figure 6D). To investigate whether URI1 induces autophagy via AMPK/FOXO3 pathway under the condotion of irradiation, cells were separately treated with URI1i plasmid or AICAR or DOX and subjected to irradiation. Results showed that depletion of URI1 downregulated the phosphorylation of AMPK and FOXO3 during irradiation, which resulting in the decreased autophagy (Figure 6E). These results indicated that URI1 suppresses ROS by activating autophagy through AMPK/FOXO3 pathway. To investigate the effect of AMPK on ROS, HepG2 cells were pre-treated with AICAR and then subjected to irradiation. Results showed that cells pre-treated with AICAR showed a significant decrease in ROS levels compared to the cells exposed to irradiation alone (Figure 6F).

## Discussion

Hepatocellular carcinoma (HCC) shows irradiation insensitivity during radiotherapy treatment 40. Accordingly, exploring the mechanism of irradiation insensitivity of HCC is significantly important. URI1 exhibits characteristics similar to those oncoproteins. It has been proved that the expression of URI1 is increased when HCC cells were exposed to irradiation and overexpression of URI1 could significantly inhibit irradiation-induced apoptosis 12. The evading apoptosis of HCC cells may result in radioresistance, which means URI1 might play an important role in the irradiation insensitivity of HCC. The damage of irradiation to cells results in dysfunctional organelle states and perturbed signaling networks, which includes intracellular ROS production 41. Excessive ROS leads to significant DNA damage and apoptosis 42. Therefore, we focused on the effect of URI1 on irradiation-induced ROS and found overexpression of URI1 could significantly suppress irradiation-induced ROS, while depletion of URI1 led to a rise of irradiation-induced ROS (Figure 1C&D).

Autophagy is a defensive mechanism 43 which maintains intracellular homeostasis by delivering cellular constituents to lysosomes, eliminating dangerous signals and recycling cytoplasmic content 44. It has been proved that autophagy can eliminate the source of oxidative stress to protect cells from oxidative damage 45 and targeting autophagy to alter cancer cells radiosensitivity can improve therapeutic efficiency 46. It suggested to us that URI1 might suppress irradiation-induced ROS through autophagy. Therefore, we explored the effect of URI1 on autophagy. We examined the expression of MAP1LC3B -II which is a marker of autophagy and the number of autophagosomes. The results showed that overexpression of URI1 significantly improved the expression of MAP1LC3B -II and the number of autophagosomes in HepG2 cells (Figure 2B-D), which meant URI1 activated cellular autophagy. Then, we explored the effect of autophagy on ROS during irradiation. We used rapamycin to activate autophagy or 3-MA to inhibit autophagy and then subjected to irradiation. Our data showed that the ROS level was significantly increased when autophagy was inhibited by 3-MA and the ROS level led to a decline when autophagy was activated by rapamycin (Figure 3C-E). It indicated that autophagy eliminated irradiation-induced ROS in HepG2 cells. Based on these data, we explored whether URI1 suppressed ROS through autophagy under the condition of irradiation. Our data showed that overexpression of URI1 further suppressed the irradiation-induced ROS when autophagy was activated by rapamycin and depletion of URI1 also further increased irradiation-induced ROS when autophagy was inhibited by 3-MA (Figure 4D&E). It indicated that the URI1-activated autophagy suppressed irradiation-induced ROS, which explained how URI1 exerted cytoprotective effects and maintained redox homeostasis during irradiation.

As a transcription factor, forkhead box O3 (FOXO3) is known as a regulator of autophagy 47. A majority of autophagy-related genes are FOXO3 transcriptional targets 48. A recent finding showed that FOXO3 activated autophagy to maintain redox homeostasis in human mesenchymal stem cells 49. Therefore, we explored whether URI1 activated autophagy via FOXO3. We found that URI1 increased the expression of FOXO3 in mRNA and protein levels (Figure 5) and the increased-FOXO3 could activate autophagy. As an upstream gene of FOXO3, AMP activated protein kinases (AMPK) plays a central role in cellular energy metabolism homeostasis 50. Moreover, AMPK functions as the upstream of mTOR to regulate the phosphorylation of mTOR, which is a typical signaling pathway in regulating autophagy 51. It has been reported that URI1 could regulate mTOR-dependent transcription signaling pathways 52. Therefore, we explored whether URI1 activated autophagy by regulating the activity of AMPK. Results showed that URI1 activated AMPK by promoting the phosphorylation of AMPKα at the Thr172 residue and the activated-AMPK promoted the the phosphorylation of FOXO3 at the Ser253 residue. The activated AMPK/FOXO3 induced autophagy, which meant URI1 activated autophagy through AMPK/FOXO3 signaling pathway. These results showed the specific mechanism of URI1-induced autophagy. However, it is regrettable that the concrete way of URI1 activated AMPK signaling pathway still remains unclear. Increasing evidences show that URI1 plays several regulatory roles in different cellular compartments 53. In the nucleus, URI1 acts as a transcriptional regulator through binding to RNA polymerase II (polII) 54; in the mitochondria, URI1 acts as a mitochondrial substrate for ribosomal protein S6 kinase 1(S6K1) to promote cells growth and survival by integrating nutrient and growth factor signaling 55. Moreover, URI1 can function as a binding protein to interact with serine protcin phosphatase 2A (PP2A) and play an important role in regulating the phosphorylation level of proteins through recruiting related phosphatase 56; in the cytoplasm, URI1 could act as a chaperone-like protein to regulate nutrient sensitive 57. Therefore, the function of URI1 needs more attention and will be the focus of our further research.

In conclusion, this study focused on the molecular mechanism of irradiation insensitivity of HCC and explored how URI1 acted as an oncogene to exert cytoprotective effects during irradiation. It proved that URI1-activated autophagy suppressed ROS levels and maintained redox homeostasis which could protect HCC from irradiation-induced oxidative damage. Our data demonstrated that URI1 activated autophagy through AMPK/FOXO3 signaling pathway. These findings elucidate a novel molecular mechanism of irradiation insensitivity of HCC.

## Figures and Tables

**Figure 7:**
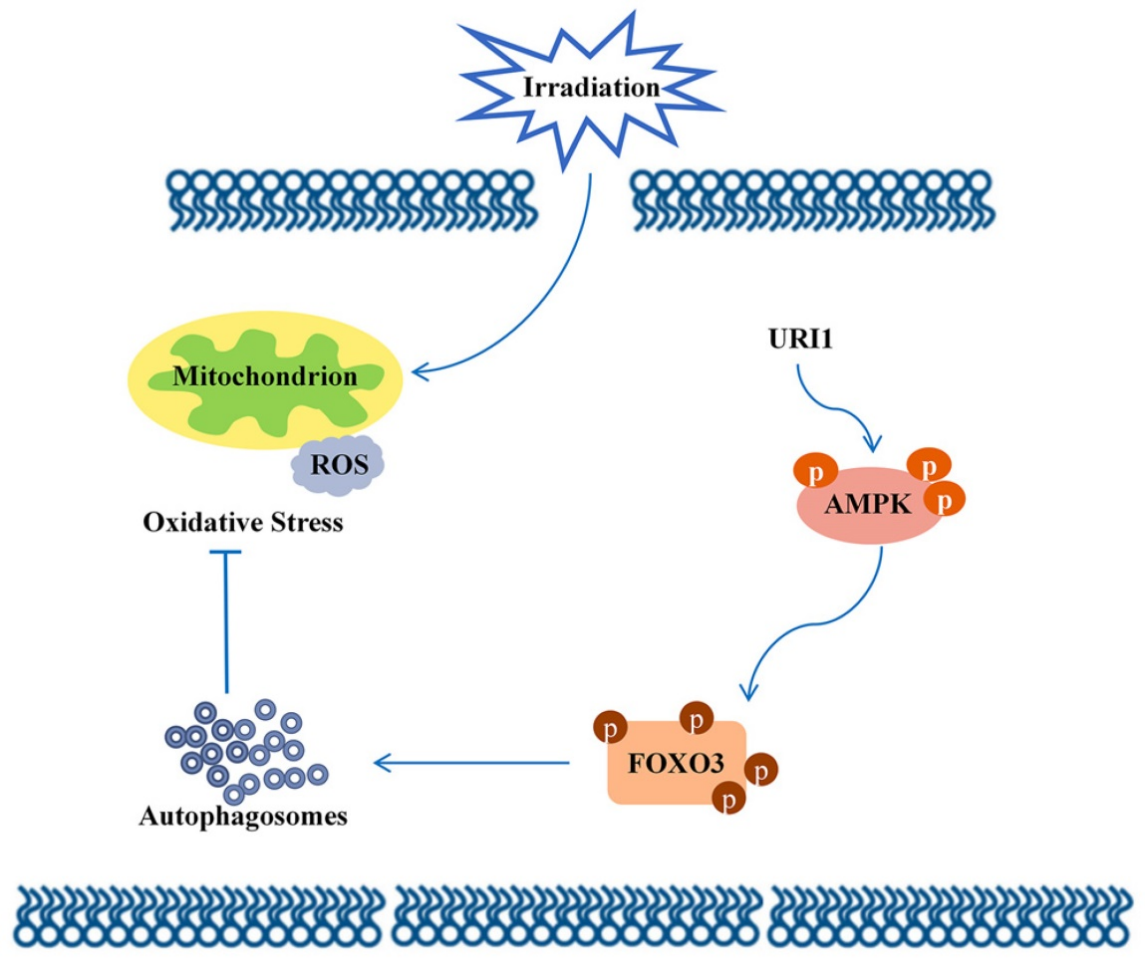
Molecular mechanism diagram.
